# Stable and discriminating features are predictive of cancer presence and Gleason grade in radical prostatectomy specimens: a multi-site study

**DOI:** 10.1038/s41598-018-33026-5

**Published:** 2018-10-08

**Authors:** Patrick Leo, Robin Elliott, Natalie N. C. Shih, Sanjay Gupta, Michael Feldman, Anant Madabhushi

**Affiliations:** 1 Case Western Reserve University, Dept. of Biomedical Engineering, Cleveland, OH 44106 United States; 2 Case Western Reserve University, Dept. of Pathology, Cleveland, OH 44106 United States; 3 University of Pennsylvania, Dept. of Pathology, Philadelphia, PA 19104 United States; 4 Case Western Reserve University, Dept. of Urology, Cleveland, OH 44106 United States

## Abstract

Site variation in fixation, staining, and scanning can confound automated tissue based image classifiers for disease characterization. In this study we incorporated stability into four feature selection methods for identifying the most robust and discriminating features for two prostate histopathology classification tasks. We evaluated 242 morphology features from N = 212 prostatectomy specimens from four sites for automated cancer detection and grading. We quantified instability as the rate of significant cross-site feature differences. We mapped feature stability and discriminability using 188 non-cancerous and 210 cancerous regions via 3-fold cross validation, then held one site out, creating independent training and testing sets. In training, one feature set was selected only for discriminability, another for discriminability and stability. We trained a classifier with each feature set, testing on the hold out site. Experiments were repeated with 117 Gleason grade 3 and 112 grade 4 regions. Stability was calculated across non-cancerous regions. Gland shape features yielded the best stability and area under the receiver operating curve (AUC) trade-off while co-occurrence texture features were generally unstable. Our stability-informed method produced a cancer detection AUC of 0.98 ± 0.05 and increased average Gleason grading AUC by 4.38%. Color normalization of the images tended to exacerbate feature instability.

## Introduction

There has been a great deal of interest in developing image analytic and feature-based machine learning tools for diagnosis and characterization of disease on digitized pathology images^1–5^. Image feature-based approaches typically involve mining several (sometimes hundreds) features from the tissue images and then identifying the subset of features most predictive of disease presence or category (e.g. cancer grade). These features are then used to train a model to predict image or patient class based on the differences in feature value distribution between images or patients of the different categories. The presence of substantial image differences between the training and testing sets on account of specimen fixation, sectioning, mounting, staining, or digitization could result in the features identified from the training set not generalizing to the validation set. To be clinically useful, a computer aided diagnosis algorithm must be able to perform equally well on images from any laboratory and be robust to inter-site variation. Hence, the set of features identified and employed in conjunction with the machine classifier needs to be robust across multiple sources of variation.

**Figure 1:**
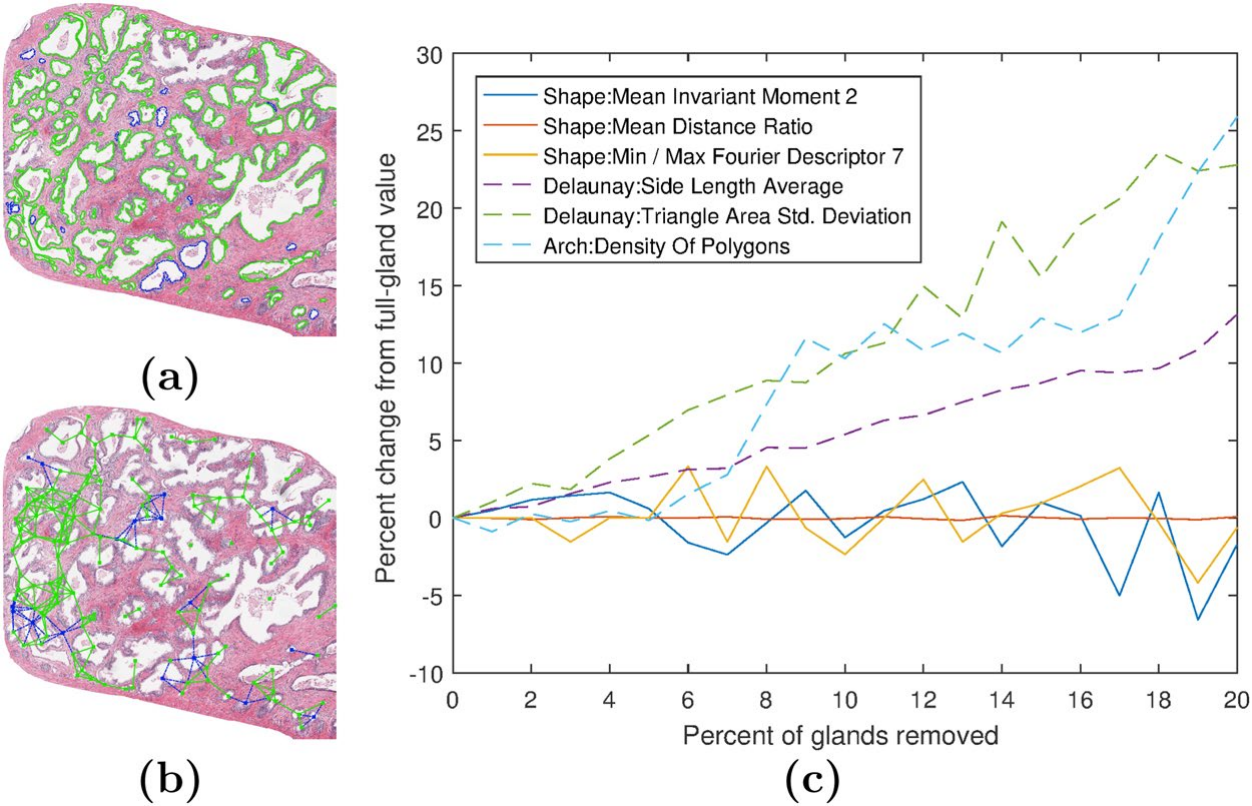
Effect of small variation in segmentation on resulting gland derived feature values. In this experiment we randomly removed a varying percentage of the total number of glands in a digitized image of a radical prostatectomy specimen in order to evaluate the corresponding effect on feature instability. (**a**) Sample benign regions of interest (ROIs) along with automatically identified gland boundaries are shown. Blue glands are those randomly selected for removal. (**b**) Gland sub-graph map, a histomorphometric feature which quantitatively captures gland architecture on the digital slide image, is illustrated. The addition of the glands with blue boundaries, though representing just 10% of the total number of glands, greatly changes the sub-graph map by connecting previously disconnected glands. Clearly, different gland detection algorithms, no matter how accurate, are likely to miss at least some percentage of glands in the image and hence choice of algorithm can substantially impact resulting features. (**c**) Plot of the percentage change in six features when removing 0 to 20% of randomly chosen glands from the region in (**a**). We systematically removed 0 to 20% of all glands in the image, in increments of 1%. For every 1% removal of glands, we ran 10 simulations for what the corresponding 6 features values might be. The averaged values for each gland removal percentage number is reported in (**c**). The values of the three most stable (solid lines) and unstable (dashed lines) features are shown in (**c**).

Prior to digitization, site-specific variation in specimen preparation can affect slide appearance. Dye batch, manufacturer, and concentration have been shown to significantly affect the slide appearance^6^. During slide digitization, factors such as the individual whole-slide scanner used, image magnification, and file compression may alter the final appearance of the image. Site-specific factors that affect image color, brightness, and contrast also affect the texture features calculated from pixel intensity values. Features derived from nuclei and glands that rely on object boundaries are also sensitive to the choice of segmentation scheme employed for extracting boundary contours^7^. Figure 1 reveals how a small variation in segmentation of individual glands can dramatically affect the resulting features.

Clearly, there is a need for both standardization of image preparation procedures and identification of features which are more and less vulnerable to instability induced by site-specific variations. One way of ensuring feature robustness is to tightly control pre-analytic sources of variation such as choice of scanner, section thickness, slide preparation, and staining concentrations. However, it is difficult enough controlling for these pre-analytic sources of variance within a single site or institution, let alone across multiple sites^6,8^. Consequently, multiple groups have attempted to standardize the acquired tissue image with color normalization and standardization algorithms^9–11^. While a number of these algorithms have shown the ability to improve color consistency within tissue compartments before and after standardization, it is less clear that this approach can adequately ameliorate all additional sources of pre-analytic variance such as choice of scanner and slice thickness. In other words, it is not clear that color standardization by itself will address the issue of reproducibility and consistency of tissue image derived feature measurements.

Since explicitly and comprehensively correcting for every source of pre-analytic variation is not tenable, another option is to identify which features are not just predictive but also stable across the multiple sources of variation. Most feature selection approaches typically rely on maximizing accuracy, area under the receiver operating characteristic curve (AUC) or some other discrimination or classification specific performance measure. Feature instability on the other hand, has not been explicitly encoded into most feature selection algorithms. This is not to suggest that the concept of feature stability has not been previously investigated in the context of image classification. Kalousis *et al*.^12^, Abeel *et al*.^13^, and Parmar *et al*.^14^ evaluated the stability of feature selection methods, defining selection methods as stable if they assigned features the same weight or rank across multiple bootstrapped folds of the training set. Yu *et al*.^15^ reviews a number of methods for calculating the similarity of two selected sets of features to quantify the agreement between feature selection methods on two datasets. These studies used datasets drawn from proteomics, genomics, text mining^12^, tissue microarrays of leukemia, prostate, colon, lymph nodes^13^, and computed tomography images of lung cancer^14^. While these approaches have the potential to help identify the appropriate feature selection approach, they do not by themselves provide any real insight on the stability of the actual features across multiple different sites. In the digital pathology space, there have been recent studies documenting sources of pre-analytic variation that influence the color of the resulting tissue scanned images^16–18^, but little by way of evaluation measures for consistency and reproducibility of tissue derived image features.

Leo *et al*.^19^ introduced two measures of feature stability, latent instability (LI) and preparation-induced instability (PI). LI captures the instability from differences in patient population in the absence of site-specific variation and is calculated by randomly splitting in half the images from a single site, and comparing the distribution of feature values in each half with the Wilcoxon rank-sum test. PI represents the rate at which a feature was observed to be significantly different in distribution between sites. While both LI and PI were employed in that study to quantitatively evaluate the relative instability associated with histomorphometric features for prostate cancer diagnosis from digital pathology tissue slide images, the study stopped short of actually using these measures to perform feature selection.

In this paper we evaluated four feature selection methods, sequential forward selection (SFS), Wilcoxon rank sum (WLCX), maximum relevance minimum redundancy (mRMR), and area under the empirical receiver operating characteristic curve (ROC), to also include feature stability across sites. Our goal was to quantitatively identify features which were both accurate and stable and, in two use cases, demonstrate a beneficial or at least non-deleterious effect on classifier performance from selecting features only from the subset of features which were stable. In the first use case we considered the problem of prostate cancer detection on surgically excised radical prostatectomy images obtained from four different sites. The second use case focused on identifying the optimal features for distinguishing Gleason grade 4 from Gleason grade 3 patterns. For both problems, the images from three sites were used for discovery and identification of the most stable and accurate features while the hold out site was used for independent validation.

In this work we primarily focused on gland derived features, using a total of 216 features relating to gland morphology and describing the global graph, lumen shape, local sub-graph, and orientation disorder^20^. Gland boundaries were obtained using the method of Nguyen *et al*.^21^. Apart from gland shape features, 26 Haralick texture features derived from pixel intensity values were also extracted to capture the textural patterns within the regions of interest. These features were chosen for their previously demonstrated performance in histopathology cancer detection and grading tasks^3,20,22–25^. For each of the two experiments, cancer detection and grading, we identified the most simultaneously stable and discriminating features across the four independent sites and also identified which feature selection scheme yielded the most consistently accurate prediction results. We also evaluated the performance of the different feature selection algorithms following application of a popular color standardization scheme to evaluate the effect of color standardization on feature instability.

## Dataset Description

A total of 212 digitized radical prostatectomy specimens were gathered from four sites as illustrated in Table 1. Each site contributed a single dataset of images. The NIH Cancer Genome Atlas yielded digitized prostatectomy images from the University of Pittsburgh and Roswell Park. No information regarding the specific scanner used for the University of Pittsburgh and Roswell Park slides was available, but the slides from the University of Pennsylvania were scanned with an Aperio CS2 and the University Hospitals (UH) Cleveland slides with an Aperio SCN400, respectively. The University of Pennsylvania slides were digitized at a 20X magnification (0.5 microns per pixel), all others were digitized at 40X (0.25 microns per pixel).

**Table 1:** Patient dataset.

Site	Patients	Cancer regions	Non-cancer regions	Grade 3 regions	Grade 4 regions
Univ. of Pennsylvania	80	80	73	39	44
Univ. of Pittsburgh	35	35	26	24	24
Roswell Park	33	33	26	28	22
UH Cleveland	64	62	63	26	22

**Figure 2:**
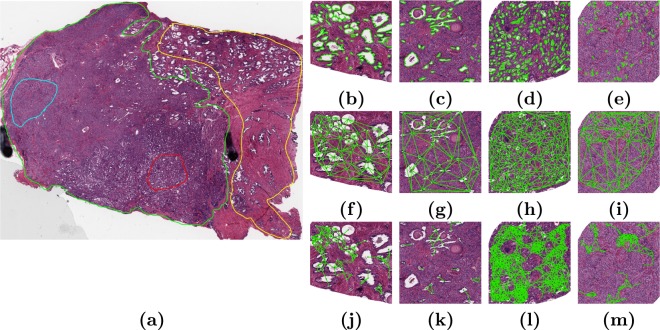
Annotated digitized radical prostatectomy image with corresponding feature maps of three feature families (shape, global graph, and sub-graph). (**a**) Radical prostatectomy specimen with expert pathologist annotations for non-cancerous (yellow), cancerous (green), homogeneous Gleason 3 (red) and homogeneous Gleason 4 (blue) regions. (**b**,**f**,**j**) Benign, (**c**,**g**,**k**) cancerous, (**d**,**h**,**l**) Gleason 3, and (**e**,**i**,**m**) Gleason 4 regions of interest. Visualization of (**b**–**e**) automated segmentation using the method of Nguyen *et al*.^21^. From these segmentations, gland area, perimeter, and boundary descriptors are calculated. (**f**–**i**) Delaunay triangulation, from which measures of gland arrangement and density such as average and standard deviation of edge length and polygon area are extracted. (**j**–**m**) Sub-graphs, local graphs of gland architecture from which measurements relating to gland packing, average degree and radius of graphs, number of isolated nodes, and clustering descriptors are extracted.

Every image was annotated by an expert pathologist as corresponding to cancerous, non-cancerous, homogeneous Gleason grade 3, and homogeneous Gleason grade 4 regions. Non-cancerous regions were identified by an expert pathologist as those locations on the digitized surgically resected tissue image not containing any cancer. Two images from UH Cleveland contained no cancerous regions, though these samples were taken from patients with confirmed prostate cancer. Thus these two images contributed only non-cancerous regions to this study. Where the pathologist identified multiple regions of a single class on an image, the largest region was used for subsequent analysis and interrogation. Figure 2 shows a representative annotated image of a surgically excised prostatectomy specimen with pathologist annotations of the different disease categories.

## Methods

### Gland Segmentation

Glands were automatically segmented from annotated ROIs using the method of Nguyen *et al*.^21^. All gland segmentations were performed on images that had been downsized to an equivalent 5X magnification. Images were further downsized to 1.25X for feature extraction. Each annotated region of interest was processed as a separate image. In the Nguyen segmentation method, k-means clustering with *k* = 4 is performed on 10,000 randomly chosen pixels from throughout the image in the three dimensional RGB space. The four centroids are then assigned the labels nuclei, cytoplasm, stroma, and lumen. Based on these four centroids, every pixel in the image is assigned a label based on the centroid it is closest to in the RGB space. The area around pixels identified as lumen are segmented and their boundaries smoothed. From the gland boundaries, the centroid of the gland is identified.

### Feature extraction

The five feature families used in this study were chosen based on evidence in the literature with regard to their ability to identify^20^ and grade^26^ prostate cancer.

#### Global graph

The global graph features are derived from gland centroid coordinates. This family consists of 51 descriptors of gland arrangement and density derived measurements from the edges and polygons of Voronoi and Delaunay maps as well as architectural measurements such as mean and standard deviation of distance to nearest three, five, seven neighbors and number of neighbors in a 40, 60, 80, and 100 micron radius of each gland^27^.

#### Gland shape

The shape features depend on the gland boundary points and capture gland appearance and boundary characteristics^27^. The 25 shape measurements include invariant and Fourier descriptors of boundary points, fractal dimension, smoothness, area, and perimeter. The mean, median, standard deviation, and minimum/maximum ratio of these 25 measurements are all calculated, for a total of 100 features.

#### Gland orientation disorder

The 39 orientation disorder features quantitatively describe how chaotic the glands in an image appear and are derived from an 18 × 18 co-occurrence matrix, corresponding to gland orientation angles binned in intervals of 10 degrees from 0 to 180 degrees. Lee *et al*.^20^ showed that gland disorder was associated with increased risk of 5-year biochemical recurrence. Gland orientation is determined from the first principal component direction of the gland boundary points. Every gland’s orientation angle is the difference between the gland’s first principal component vector and a constant arbitrary vector. The co-occurrence matrix is then populated with entry (*i*, *j*) equaling the number of times a gland of orientation *i* was found in the same sub-graph as a gland of orientation *j*. The orientation disorder features use gland centroid coordinates for constructing neighborhood sub-graphs and gland boundary points for calculating orientation. A co-occurrence matrix is constructed for every sub-graph in an image and measures of entropy, variance, and energy are extracted from the matrix.

#### Sub-graph

26 sub-graph^28^ features describe the local gland arrangement, packing, and clustering, by connecting nearby glands together into a graph based on the gland centroid coordinates. The sub-graph features include sub-graph radius, eccentricity, clustering, path length, ratio of glands in the largest sub-graph to total number of glands, and percentage of glands which are isolated.

#### Haralick

Unlike the aforementioned features, the 39 Haralick texture features^25^ depend on pixel intensity values. The entire annotated region of interest is converted to a grayscale image and all pixels in the region are used for feature calculations. These features are drawn from a co-occurrence matrix describing how often pixels of various intensities are found near pixels of another intensity. The Haralick features describe the texture, edges, gradients, spots, and homogeneity of the image. As with the gland orientation disorder features, the entropy, variance, and energy are calculated from the co-occurrence matrix.

### Quantifying feature instability and feature performance

In this study our goal was to investigate if there were features which were both highly accurate and highly stable. Feature instability was calculated using the non-cancerous regions of radical prostatectomy specimens from each site. Differences in feature values between non-cancerous regions between sites were considered evidence of site-linked instability since it was considered unlikely that the morphology and texture of these non-cancerous regions would vary across sites without site-specific confounding effects.

To determine the relationship between feature instability and discriminability, we first calculated the AUC for each of the 242 features in the study. A single feature at a time was used for classification in the cancer detection and Gleason grading tasks. Feature AUC was calculated with 100 iterations of 3-fold cross validation using a linear discriminant analysis (LDA) classifier. Classification was performed separately for each site and the mean AUC across the four sites was used to generate the final AUC for each feature. The PI associated with each feature was then computed across all the non-cancerous regions for the the four sites. Each feature had two values, PI across non-cancerous regions and mean AUC. Thus each feature was defined by a unique position in the PI-AUC space.

### Feature selection schemes

We employed four feature selection schemes, sequential forward selection (SFS)^29^, Wilcoxon rank-sum (WLCX)^30^, maximum relevance minimum redundancy (mRMR)^31^, and area under the empirical receiver operating characteristic curve^32^ (ROC). These feature selection methods were selected to represent four different approaches to identifying discriminating features. The approaches used are: iterative selection (SFS), pure significance testing (WLCX), discriminability and redundancy testing (mRMR), and pure accuracy testing (ROC).

#### Sequential forward selection

SFS iteratively adds features to the selected set, starting with an empty set. In every iteration, the feature which maximizes the objective function when added to the already selected set is added to the selected set. Our objective function was AUC in 10 iterations of 3-fold cross validation with a quadratic discriminant analysis (QDA) classifier.

#### Wilcoxon rank-sum

The Wilcoxon rank-sum test seeks to evaluate the difference in medians between two distributions. Given a set of feature values from studies, the first step in scoring the feature is to rank the values from least to greatest. Using the ranks and labels of each study (cancer/non-cancer or Gleason 3/Gleason 4), the sum of the ranks of studies from each class is computed. Selected features were those which had the largest difference in the sum of ranks between the two classes.

#### Maximum relevance minimum redundancy

mRMR considers both discriminability and independence when selecting features. Beginning from an empty set, the feature with the maximum mutual information with the target class and minimum mutual information with already selected features is added to the selected set.

#### Empirical receiver operating characteristic curve

The ROC method constructs a classifier for each feature-based only on a feature value threshold. By sweeping the threshold from the minimum to maximum feature value and using the class labels, a receiver operating characteristic curve can be constructed. If the distributions of a feature’s values in the two classes have a low degree of overlap, that feature will have a high AUC. The features with the greatest AUC were selected. This approach does not correct for features that might be highly correlated with respect to each other.

### Novel stability-informed feature selection

For each of the afore-mentioned feature selection methods we sought to evaluate whether the inclusion of stability would be non-inferior to and potentially improve classification performance over not including stability, for each of two prostate cancer diagnosis tasks considered in this study. When selecting features for stability as well as discriminability, we excluded from consideration every feature which exhibited a PI above a predetermined threshold (PI = 0.25) in the training set. A PI of 0.25 indicates that a feature was significantly different between non-cancerous regions of the sites in the training set in 25% of comparisons. We then applied the four different feature selection methods (SFS, WLCX, mRMR, ROC) to select features from amongst the set of features with a PI < 0.25. This is the discriminability-and-stability-selected feature selection method $F{S}_{sd}^{\theta }$, $\theta \in \{SFS,WLCX,mRMR,ROC\}$. To evaluate the added value of inclusion of the stability measure, we also used evaluated the four original feature selection methods without the inclusion of the stability constraint on the entire set of 242 features. These approaches were designated as $F{S}_{d}$.

To evaluate $F{S}_{sd}^{\theta }$ we performed hold-one-site-out classification in which three sites were used to train a model which was then tested on the hold out site. Feature PI was calculated across the non-cancerous regions of the three site cohorts of the training set. Feature discriminability was calculated on all patients in the training set. Using the top 5 features, four classifiers, LDA, QDA, support vector machine (SVM) and random forest (RF) were trained on the training set and applied to the images from the hold out site. Classifiers were selected to span from low-complexity purely linear (LDA) to high-complexity non-linear (RF) with QDA and SVM falling between those two extremes. The model resulting from a combination of a feature selection method and a classifier is expressed as $F{S}_{sd}^{\theta ,\kappa }$, $\theta \in \{SFS,WLCX,mRMR,ROC\}$, $\kappa \in \{LDA,QDA,SVM,RF\}$ for discriminability-and-stability selected model and $F{S}_{d}^{\theta ,\kappa }$ for discriminability-selected models.

### Performance measures

The two metrics we employed to evaluate the features were PI and AUC, both of which range from 0 to 1. Since PI measures feature instability, a value of 0 corresponds to an ideal feature which is never affected by site variation and a value of 1 corresponds to a feature which is never the same between sites. AUC describes the performance of a classifier in terms of its ratio of true positives to false positives at every classification-confidence threshold. An AUC of 0.5 is equal to guessing, where a true positive is just as likely as a false positive, and an AUC of 1 is an ideal classifier which classified every input correctly.

The ideal feature would have a PI of 0 and an AUC of 1, meaning it would never vary between sites and would perfectly separate the two classes of interest. The best features are those which occupy the low-PI, high-AUC space while the worst features are found in the high-PI, low-AUC space.

### Experiment 1: Cancerous vs. non-cancerous region classification

Our first experiment was to identify stable and discriminating features and to evaluate whether $F{S}_{sd}^{\theta }\theta \in \{SFS,WLCX,mRMR,ROC\}$ might allow for improvement of cross-site classification performance in the context of a cancer detection problem. In the training set, the non-cancerous regions were used to calculate feature instability and feature discriminability, which in turn was based on the difference between cancerous and non-cancerous regions.

### Experiment 2: Gleason 3 vs. Gleason 4 region classification

Our second experiment sought to identify stable and accurate features and use $F{S}_{sd}^{\theta }$ in classifying homogeneous Gleason 3 and Gleason 4 regions. Homogeneous regions were identified as those that comprised no less than 95% pure Gleason grade 3 or 4 patterns. The total of 229 homogeneous Gleason 3 or Gleason 4 regions were drawn from 157 patients. The 55 patients without a homogeneous region of either type were not used for calculating feature discriminability. Non-cancerous regions from all patients were used to calculate feature instability. The non-cancerous regions were used to calculate instability in both experiments since those regions were both numerous and unlikely to be affected by other factors such as tumor grade.

### Experiment 3: Color normalization

Our third experiment attempted to evaluate whether color normalization could sufficiently reconcile the site-specific variations induced by different laboratories and institutions. In other words the goal was to evaluate whether color normalization by itself could potentially obviate the need for considerations of feature stability. The effect of color normalization was evaluated by comparing feature PI values before and after normalization. Our chosen method was that of Macenko *et al*.^11^. In this method, the optical density of every pixel is identified. Background pixels, which were pixels with an optical density less than 0.15, were removed and this optical density matrix (OD) was then factored into its components *V* and *S* with non-negative matrix factorization, such that *OD* = *VS*. The singular value decomposition (SVD) of the (*V*, *S*) points was found and the angles between each point and the first SVD direction was calculated. The 1st and 99th percentiles of these angles represented the ideal stain vectors. Color deconvolution was performed on the RGB image with the two ideal stain vectors to isolate each stain in the image. Pixels were assigned to the hematoxylin or eosin class based on which stain intensity was higher in the pixel. The intensity histograms of each stain were then normalized to a template and the deconvolved images were transformed back into an RGB image. Glands were then segmented on the normalized images, and feature instability was recalculated from the non-cancerous regions. A reduction in a feature’s PI following color normalization would suggest that color normalization had ameliorated the variations in image appearance across sites, that is the original image from which the feature was derived

## Results

### Results of Experiment 1: Cancer vs. non-cancer

**Figure 3:**
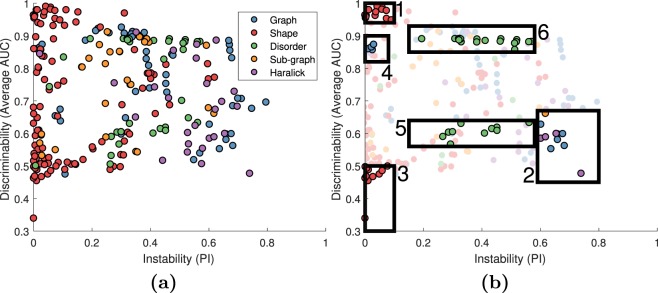
(**a**) PI-AUC plot for 242 features for 212 studies across four sites for cancer vs. non-cancer classification. Each feature is represented by a dot, color coded according to feature family. On the X-axis is the PI value for each feature in the non-cancerous regions of the four sites. On the Y-axis is the feature’s AUC averaged across 100 iterations of 3-fold cross validation across all patients from all four sites. Shown are 242 features from the global graph (blue), shape (red), disorder (green), sub-graph (yellow), and Haralick (purple) feature families. The optimal high-AUC, low-PI space is dominated by shape features while the low-AUC, high-PI space comprises Haralick and global graph features. (**b**) ROIs corresponding to features occupying different regions in the PI-AUC space, see Table 2.

Figure 3 shows the PI-AUC space for 242 features for N = 212 patients from all four sites for cancerous vs. non-cancerous region classification. The results shown in Fig. 3 were not used in selecting features as Fig. 3 includes all the data, without a training/testing split. Instead, Fig. 3 provides insight into where various features and families lie in the PI-AUC space. The best performing features were all shape features while the most unstable and least discriminating features were all Haralick and global graph features. There was no apparent correlation between feature AUC and instability either in the whole feature set or in any feature family.

Many features have an AUC close to 1. Even so, there is great variation both between and within the feature families in terms of feature performance (see Table 2). The ROIs shown in Table 2 indicate that features found in similar areas of the PI-AUC plot appear to be correlated, suggesting that the AUC-stability trends are related for features within a specific feature family.

**Table 2:** 70 features found in ROIs of the PI-AUC space shown in Fig. 3(b).

**1: High AUC** (>**0**.**94**), **low PI** (<**0**.**1**)	**2: Low AUC** (<**0**.**67**), **high PI** (>**0**.**59**)
(S) Mean distance ratio	(G) Voronoi area std. deviation
(S) Mean smoothness	(G) Voronoi chord std. deviation
(S) Mean invariant moment 1	(G) Delaunay side length min/max
(S) Mean invariant moment 2	(G) Delaunay triangle area min/max
(S) Mean invariant moment 4	(G) Delaunay triangle area std. deviation
(S) Std. deviation distance ratio	(G) Voronoi polygon area
(S) Std. deviation smoothness	(SG) Number of end nodes
(S) Std. deviation Fourier descriptor 1	(H) Mean average intensity
(S) Std. deviation Fourier descriptor 2	(H) Mean entropy
(S) Std. deviation Fourier descriptor 3	(H) Std. deviation entropy
(S) Std. deviation Fourier descriptor 4	(H) Mean information measure 1
(S) Std. deviation Fourier descriptor 5	**4: High AUC** (>**0**.**82**), **low PI** (<**0**.**05**) **graph**
(S) Std. deviation Fourier descriptor 8	(G) Std. deviation neighbors in 40 micron radius
(S) Mean invariant moment 6	(G) Std. deviation neighbors in 60 micron radius
**3: Low AUC** (<**0**.**5**), **low PI** (<**0**.**1**) **shape**	(G) Std. deviation neighbors in 80 micron radius
(S) Mean fractal dimension	(G) Std. deviation neighbors in 100 micron radius
(S) Mean Fourier descriptor 2	**6: High AUC** (>**0**.**85**) **disorder**
(S) Mean Fourier descriptor 5	(D) Std. deviation tensor contrast energy
(S) Median invariant moment 7	(D) Mean tensor contrast inverse moment
(S) Median fractal dimension	(D) Std. deviation tensor contrast inverse moment
(S) Median Fourier descriptor 1	(D) Mean tensor contrast average
(S) Median Fourier descriptor 2	(D) Std. deviation tensor contrast average
(S) Min/max invariant moment 6	(D) Std. deviation tensor contrast variance
(S) Min/max invariant moment 7	(D) Mean tensor contrast entropy
(S) Min/max Fourier descriptor 7	(D) Mean tensor contrast entropy
(S) Min/max Fourier descriptor 9	(D) Std. deviation tensor intensity average
**5: Mid AUC** (<**0**.**64**, >**0**.**56**) **disorder**	(D) Mean tensor contrast entropy
(D) Range tensor contrast energy	(D) Std. deviation tensor intensity variance
(D) Range tensor contrast inverse moment	(D) Mean tensor intensity entropy
(D) Range tensor contrast variance	(D) Std. deviation tensor intensity entropy
(D) Range tensor contrast entropy	(D) Mean tensor entropy
(D) Range tensor intensity average	(D) Mean tensor energy
(D) Range tensor intensity variance	(D) Std. deviation tensor energy
(D) Range tensor intensity entropy	(D) Mean tensor correlation
(D) Range tensor entropy	(D) Std. deviation tensor correlation
(D) Range tensor correlation	(D) Mean tensor information measure 2
(D) Range tensor information measure 1	(D) Std. deviation tensor information measure 2

Feature PI and AUC values were found by using all 212 patients from all four sites. PI is calculated across non-cancerous regions and AUC is the mean from 100 iterations of 3-fold cross validation for the cancer vs. non-cancer classification task. Cross validation was performed independently for each site.

In cross-site hold-one-site-out classification, *FS*_*sd*_ performed nearly as well as *FS*_*d*_, with both having an average AUC of 0.98. Table 3 shows detailed results of the performance of each feature selection scheme and classifier.

**Table 3:** Mean (standard deviation) of AUC for the cancer vs. non-cancer classification problem across the four hold-one-site-out folds with $F{S}_{sd}^{\theta ,\kappa }$ and $F{S}_{d}^{\theta ,\kappa }$ $\theta \in \{SFS,WLCX,mRMR,ROC\}$, $\kappa \in \{LDA,QDA,SVM,RF\}$.

	SFS			WLCX		
	*FS*_*sd*_	*FS*_*d*_	% Improvement	*FS*_*sd*_	*FS*_*d*_	% Improvement
LDA	0.99 (0.01)	0.99 (0.01)	−0.15	0.96 (0.04)	0.97 (0.04)	−0.22
QDA	0.98 (0.02)	0.99 (0.02)	−0.11	0.95 (0.04)	0.96 (0.05)	−1.18
SVM	0.98 (0.01)	0.99 (0.01)	−0.68	0.96 (0.03)	0.97 (0.04)	−0.36
RF	0.98 (0.02)	0.99 (0.01)	−0.84	0.95 (0.03)	0.96 (0.03)	−1.63
	**mRMR**			**ROC**		
	***FS***_***sd***_	***FS***_***d***_	**% Improvement**	***FS***_***sd***_	***FS***_***d***_	**% Improvement**
LDA	0.99 (0.01)	0.99 (0.01)	−0.37	0.99 (0.01)	0.99 (0.01)	0.00
QDA	0.96 (0.04)	0.98 (0.02)	−2.32	0.98 (0.03)	0.98 (0.05)	0.00
SVM	0.97 (0.02)	0.99 (0.01)	−1.50	0.98 (0.02)	0.98 (0.01)	−0.03
RF	0.99 (0.01)	0.99 (0.01)	**0**.**16**	0.98 (0.00)	0.99 (0.00)	−0.66

For each classifier model, the top 5 most stable and discriminating or most discriminating features were employed for constructing $F{S}_{sd}$ and $F{S}_{d}$ respectively. For every feature selection-classification pair four models were trained and validated, one model for every possible combination of three of the four sites. The three chosen sites were combined and used for training and the held out site was used for validation. The improvement between $F{S}_{sd}$ and $F{S}_{d}$ is shown. A positive improvement indicates that $F{S}_{sd}$ outperformed $F{S}_{d}$. Note that for this particular problem, the prediction AUC for all models were very high, nearly perfect in most cases.

### Results of Experiment 2: Gleason 3 vs. Gleason 4 classification

**Figure 4:**
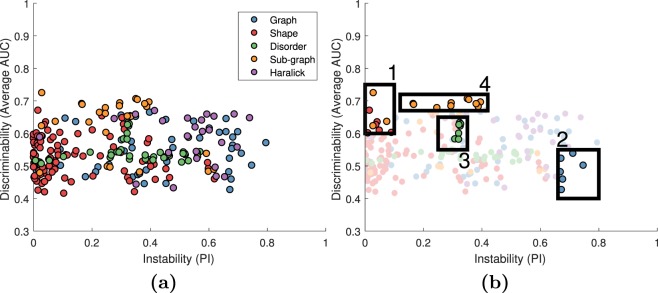
(**a**) PI-AUC plot for 242 features for 157 studies across four sites for Gleason 3 vs. Gleason 4 classification. Each feature is represented by a dot, color coded according to feature family. On the X-axis is the PI value for each feature in the non-cancerous regions of the four sites. On the Y-axis is the feature’s AUC averaged across 100 iterations of 3-fold cross validation across all patients from all four sites. Shown are 242 features from the global graph (blue), shape (red), disorder (green), sub-graph (yellow), and Haralick (purple) feature families. The optimal high-AUC, low-PI space comprises shape and sub-graph features while the low-AUC, high-PI space comprises chiefly Haralick and global graph features (**b**) ROIs corresponding to features occupying different regions in the PI-AUC space, see Table 4.

Figure 4 shows the PI-AUC space for 242 features for N = 157 patients from all four sites for Gleason 3 vs. Gleason 4 classification. As in Fig. 3, instability was calculated across the 188 non-cancerous regions. Between Figs 3 and 4, features have the same PI but different AUC values. Unlike for Experiment 1, the most stable features were not always the most accurate. The best and worst features are shown in Table 4.

**Table 4:** 32 features found in ROIs of the PI-AUC space shown in Fig. 4(b).

**1: High AUC** (>**0**.**6**), **low PI** (<**0**.**1**)	**2: Low AUC** (<**0**.**55**), **high PI** (>**0**.**66**)
(S) Mean invariant moment 1	(G) Voronoi perimeter average
(S) Std. deviation Fourier descriptor 2	(G) Delaunay side length std. deviation
(S) Std. deviation Fourier descriptor 3	(G) Delaunay side length average
(S) Median invariant moment 1	(G) Delaunay triangle area std. deviation
(S) Median invariant moment 6	(G) Delaunay triangle area average
(S) Median fractal dimension	(G) Delaunay triangle area disorder
(SG) Mean of edge length	**4: High AUC** (>**0**.**6**), **medium PI** (>**0**.**1**)
(SG) Skewness of edge length	(SG) Average eccentricity
(SG) Kurtosis of edge length	(SG) Diameter
**3: High AUC** (>**0**.**55**) **disorder**	(SG) Radius
(D) Mean tensor contrast inverse moment	(SG) Average eccentricity 90th percentile
(D) Mean tensor contrast entropy	(SG) Diameter 90th percentile
(D) Mean tensor intensity entropy	(SG) Radius 90th percentile
(D) Mean tensor entropy	(SG) Average path length
(D) Mean tensor energy	(SG) Clustering coefficient C
(D) Mean tensor correlation	(SG) Clustering coefficient D
(D) Mean tensor information measure 2	(SG) Clustering coefficient E

Feature PI values were found by using all 188 non-cancerous regions from all four sites. PI is calculated across non-cancerous regions and AUC is the mean from 100 iterations of 3-fold cross validation for the Gleason 3 vs. Gleason 4 classification task. Cross validation was performed independently for each site. The family of each feature is indicated as graph (G), shape (S), disorder (D), sub-graph (SG) or Haralick (H).

In terms of classification, *FS*_*sd*_ outperformed *FS*_*d*_ in 13 of 16 cases, (Table 5). Mean AUC was 0.74 ± 0.08 with $F{S}_{sd}^{\theta ,\kappa }$ and $0.71\pm 0.10$ with $F{S}_{d}^{\theta ,\kappa }$ $\theta \in \{SFS,WLCX,mRMR,ROC\},\kappa \in \{LDA,QDA,SVM,RF\}$. The average AUC improvement of $F{S}_{sd}$ over $F{S}_{d}$ was 4.38%. $F{S}_{sd}$ was not universally superior to $F{S}_{d}$, though overall every classifier and every feature selection method showed a net benefit from including stability. In the 81% of cases where $F{S}_{sd}$ improved AUC over $F{S}_{d}$ it did so by an average of 5.92%. SFS and WLCX were the feature selection schemes which benefited most with $F{S}_{sd}$, with average gains of 9.64% and 4.99% respectively. QDA and SVM were the classifiers which benefited most from $F{S}_{sd}$, with gains of 6.05% and 5.88% respectively.

**Table 5:** Mean (standard deviation) of AUC for the Gleason 3 vs. Gleason 4 classification problem across the four hold-one-site-out folds with $F{S}_{sd}^{\theta ,\kappa }$ and $F{S}_{d}^{\theta ,\kappa }$ $\theta \in \{SFS,WLCX,mRMR,ROC\}$, $\kappa \in \{LDA,QDA,SVM,RF\}$.

	SFS			WLCX		
	*FS*_*sd*_	*FS*_*d*_	% Improvement	*FS*_*sd*_	*FS*_*d*_	% Improvement
LDA	0.75 (0.07)	0.67 (0.06)	**11**.**71**	0.74 (0.05)	0.71 (0.10)	**4**.**28**
QDA	0.77 (0.04)	0.69 (0.09)	**11**.**58**	0.70 (0.06)	0.69 (0.06)	**1**.**91**
SVM	0.71 (0.08)	0.65 (0.10)	**8**.**99**	0.71 (0.05)	0.67 (0.09)	**6**.**93**
RF	0.72 (0.06)	0.68 (0.10)	**6**.**29**	0.76 (0.04)	0.71 (0.07)	**6**.**84**
	**mRMR**			**ROC**		
	***FS***_***sd***_	***FS***_***d***_	**% Improvement**	***FS***_***sd***_	***FS***_***d***_	**% Improvement**
LDA	0.71 (0.03)	0.71 (0.02)	−0.66	0.70 (0.08)	0.70 (0.05)	−0.15
QDA	0.66 (0.06)	0.60 (0.08)	**8**.**76**	0.71 (0.05)	0.69 (0.07)	**1**.**96**
SVM	0.64 (0.06)	0.60 (0.08)	**6**.**30**	0.72 (0.06)	0.71 (0.08)	**1**.**31**
RF	0.68 (0.04)	0.72 (0.06)	−6.02	0.74 (0.03)	0.74 (0.07)	**0**.**04**

For each classifier model, the top 5 most stable and discriminating or most discriminating features were employed for constructing $F{S}_{sd}$ and $F{S}_{d}$ respectively. For every feature selection-classification pair four models were trained and validated, one model for every possible combination of three of the four sites. The three chosen sites were combined and used for training and the held out site was used for validation. The improvement between $F{S}_{sd}$ and $F{S}_{d}$ is shown. A positive improvement indicates that $F{S}_{sd}$ outperformed $F{S}_{d}$. Improvement in $F{S}_{sd}$ over $F{S}_{d}$ occurred in 13 of the 16 cases, with the average improvement in those 13 scenarios being 5.92% compared to 2.28% when $F{S}_{d}$ was superior compared to $F{S}_{sd}$.

### Results of Experiment 3: Color normalization

**Figure 5:**
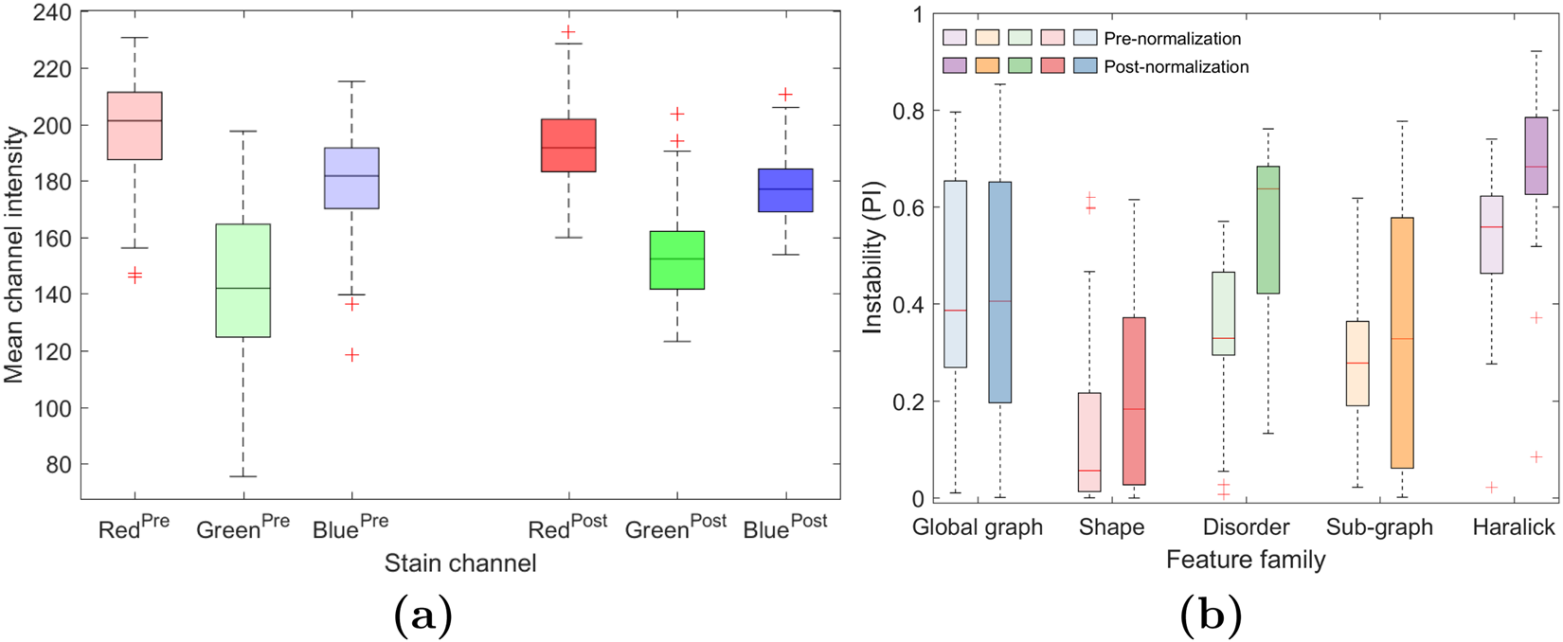
(**a**) Boxplots of mean red, green, and blue intensities in 188 non-cancerous regions pre- (left boxes) and post- (right boxes) color normalization. While our chosen normalization method works in the stain vector space, not in the RGB space, the range and variation of mean color intensities decreased after normalization, especially in the green channel. This suggests that normalization has reduced the variation in color across the images. (**b**) Feature PI by family before (lighter bars) and after (darker bars) color normalization. PI was measured across the 188 non-cancerous regions of the four sites before and after those regions were normalized. Color normalization increased instability in every feature family with an especially strong effect on the disorder features. These results suggest that color normalization is inadequate to resolve the problem of feature instability from site variation and may even worsen instability.

Color normalization increased feature instability, though feature families were affected to varying degrees as illustrated in Fig. 5. Feature instability pre- and post-normalization was calculated on all 188 non-cancerous regions from all four sites. Gland orientation disorder features showed the greatest increase in instability, with normalization raising average PI from $0.36\pm 0.15$ to $0.54\pm 0.19$ while global graph features were relatively unaffected, though it is worth noting that these features were unstable to begin with.

The 10 features which had the largest increases and decreases in their PI values on account of color normalization are shown in Table 6. Several highly accurate and highly stable shape features appeared to become more unstable by color normalization. The gland shape and disorder features showed a large increase in median PI following normalization Overall color normalization was not found to be effective in reducing feature instability. Feature instability across the 188 non-cancerous regions from all four sites was $0.34\pm 0.15$ pre-normalization and $0.43\pm 0.18$ post-normalization, a 26% increase.

**Table 6:** 20 features which showed an improvement (left column) or worsening (right column) in instability following color normalization.

Stabilized by normalization		Destabilized by normalization	
Feature	PI change	Feature	PI change
(S) Std. deviation distance ratio	−0.59	(S) Min/max Fourier descriptor 9	0.58
(S) Std. deviation area ratio	−0.59	(S) Median perimeter ratio	0.56
(G) Voronoi chord std. deviation	−0.55	(S) Median Fourier descriptor 8	0.54
(G) Voronoi perimeter std. deviation	−0.54	(S) Median invariant moment 1	0.49
(S) Std. deviation of std. deviation of distance	−0.54	(S) Min/max Fourier descriptor 1	0.46
(G) Voronoi area min/max	−0.46	(D) Mean tensor contrast average	0.45
(G) Voronoi area disorder	−0.45	(H) Mean energy	0.44
(D) Range of tensor information measure 1	−0.43	(S) Median Fourier descriptor 7	0.44
(S) Std. deviation of variance of distance	−0.40	(S) Median invariant moment 2	0.43
(G) Voronoi chord disorder	−0.37	(S) Mean invariant moment 1	0.42

N = 188 non-cancerous regions from 188 patients across all four sites were color normalized to a template and instability across the four sites was calculated before and after normalization. The 10 features with the largest absolute PI change pre- and post-normalization in each direction are shown. A negative PI change signifies a reduction in feature instability. The family of each feature is listed as graph (G), shape (S), disorder (D), sub-graph (SG), and Haralick (H).

## Discussion

The goal of this study was to determine if computer extracted histomorphometric features could be identified which were both highly accurate and highly stable for the problems of computer assisted prostate cancer detection and grading on digitized histopathology images of surgically resected specimens. To this end, we evaluated individual feature performance based on a measure of instability, preparation-induced instability score, and a measure of discriminability, AUC. We also investigated whether stability information could be used to improve the predictive performance of digital pathology classifiers for the problems of cancer detection and grading across different sites. Features which were found to be accurate and stable on the training set were used to build a model which was tested on the held out set. Lastly, we compared feature instability pre- and post-normalization. A reduction in feature instability post-normalization would imply that differences between sites manifest primarily as differences in color. Conversely, a lack of such a reduction would suggest that site-specific induced image variations were not fixable solely by color normalization alone.

In this work we employed the stability metric introduced by us in Leo *et al*.^19^ to evaluate feature robustness across different sites. Specifically we evaluated preparation-induced instability score, a measure of the observed difference between feature value distributions between sites, and employed it for improving cross-site classification performance in cancer detection and grading tasks. In our previous study^19^ we showed that instability in histomorphometric features between sites is much higher than what would be expected without inter-site effects, even when accounting for differences in patient population. However, that study did not specifically invoke the concept of stability for feature selection or for subsequent feature-based classification. In this study we used feature stability to identify stable features and select from amongst those the most stable features which would provide consistently higher predictive performance across multiple different sites.

Our first experiment, on cancerous vs. non-cancerous region classification found that the most accurate and stable features were all from the gland shape family, that Haralick features were generally unstable, and that stability-informed selection was not inferior to discriminability-only selection. The instability of the Haralick feature may owe to the large effect of sample preparation, color, and contrast on image texture. A feature’s instability score may be a reflection of how much a small change in segmentation affects the feature’s value. Such variation in segmentation performance may be due to site-induced variation in image color, contrast, and sharpness as well as tissue preservation quality and appearance. In the arrangement-variant features (e.g. features of orientation disorder or architecture), the addition or removal of just a few glands in certain locations may cause a large variation in the feature value as graphs shift. For arrangement-invariant features (e.g. those based on average gland shape), where every gland is compared with its neighbors, an added or removed gland can also affect the feature values of neighboring glands. Location dependence and neighbor dependence mean that small differences in segmentation, such as those produced by site variation, have a large effect on the value of these features.

Several other studies have produced high accuracies in cancer detection using features we found to be unstable. In Lee *et al*.^20^, gland orientation disorder features outperformed gland global graph, gland shape, and image texture features in both cancer detection and biochemical recurrence prediction in cross validation on a single site. In our study, some disorder features were highly accurate, with single-feature AUCs of 0.90, but many shape features were more accurate. Other studies have found Haralick and other texture features to be predictive in prostate cancer detection^20,26,33,34^, breast tissue cancer detection and grading^24^ and lung cancer detection^35^. Though these studies did not perform cross-site independent validation, our findings that gland disorder and Haralick features were highly unstable suggests that these features may not generalize well in cross-site tasks. It could be beneficial for future studies to perform cross-site validation as feature instability may mean that single-site results are not a good predictor of cross-site results.

The second experiment, on Gleason 3 vs. Gleason 4 classification, found that the most accurate and stable features were a mix of shape and sub-graph features, that the most unstable and inaccurate features were Haralick and global graph, and that stability-informed selection improved classification AUC in 13 of 16 cases. Our goal was not to create the best possible automated Gleason grade classifier. Instead, our goal was to attempt to showcase that considering stability in feature selection can improve cross-site classifier robustness across a range of feature selection methods and classifiers. While other studies in this area have reported accuracies above 0.80, they differed from our study in the use of nuclei features^26^, manually segmented glands^36,37^, biopsy samples^38^, or high grade vs. low grade classification^34^. Additionally, none of these studies evaluated studies from across multiple different sites. These studies found that texture^26,34,38^ and shape^36–38^ features were predictive of high versus low Gleason grade. We found shape features and some texture features to be predictive, though while shape features were highly stable, texture features were highly unstable. To our knowledge, no other study has used such a large dataset to explicitly evaluate cross-site classification of Gleason 3 and Gleason 4 regions. Gleason 3 and 4 represent the most commonly occurring low and intermediate prostate cancer patterns found^39^ and also the source of most of the inter-reader variability when it comes to grading prostate cancer pathology specimens^40^.

Interestingly, when invoking stability constrained feature selection, the resulting features tended to outperform the non-stability informed methods despite employing far fewer features. As was shown in Figs 3 and 4, many highly discriminating features were excluded based off the stability constraint. Despite the limited feature set, in Gleason grading the stability-informed method outperformed the discriminability-alone method 81% of the time with an average AUC improvement of 4.38% when the stability criterion was also invoked.

Our third experiment found that color normalization did not reduce overall feature instability, suggesting that differences between sites are not contained to variation in color. Our results are consistent with Leo *et al*.^19^ which used a different color normalization scheme and found that normalization did not resolve feature instability. There is precedent for worse standardization following color normalization. Janowczyk *et al*.^9^ compared nuclei segmentation performance under six different color normalization schemes, including the method used in this study. That study found that segmentation was not always improved by normalization and that image content affected which normalization method performed best. These findings, along with our results, suggest color normalization may not necessarily resolve the problems of feature stability on account of variations induced by different sites and labs. These findings may be specific to the color normalization method chosen, it is possible that other color normalization methods would have produced different results.

The lack of stability improvement following color normalization suggests that fixation, preservation, and mounting may cause differences in morphology that extend beyond inducing just differences in color appearance. A site that injects formalin into tissue immediately after surgery will produce tissue with less autolysis than a site that just places the tissue in a formalin bath, a difference which may affect the quantitative descriptors extracted from that tissue. Immunohistochemical expression and quality of tissue DNA have been shown to be affected by formalin injection and excessively long fixation time^41^. Though these factors have not been shown to alter the visual appearance of the tissue in the judgment of human observers, QH methods may be more affected by these subtle changes.

Limitations of this study include that we examined only one gland segmentation method, used only two types of features, gland morphology and Haralick texture, had one model training/validation division, hold-one-site-out, did not directly integrate stability into feature selection, and used only one color normalization method. Because our features were extracted only from gland lumen, regions of the Gleason 4 tissue with fused glands without lumen were not fully characterized by our features. These regions contributed to feature values only through the absence of lumen and the resulting effects on features of gland arrangement and density. Additionally, while our annotations were as precise as possible, any Gleason 3 glands in the Gleason 4 regions would contribute to the feature values of those regions. Methods for characterizing glands that do not rely on luminal analysis may better capture the morphology of Gleason 4 regions. Future work in this area will use other methods of incorporating stability into feature selection in ways beyond a simple threshold and examining the utility of stability information for other classification tasks in other problems in digital pathology.

In this study we demonstrated a novel method for identifying features which are both discriminating and stable across site variation. We then showed that our method is useful for problems of cancer detection and grading and can improve model performance on independent validation sets. Feature stability is a critical and under-investigated component in the development of computer aided diagnosis systems. By leveraging stability information from multiple sites and incorporating that information into feature selection it may be possible to mitigate the effects of inter-site variation. Given the difficulty in standardizing laboratory procedures and equipment and the lack of success of color normalization methods in reducing feature instability in this study, clinically useful classifiers may need to consider feature instability in the model training process.
